# The convergence rate of the proximal alternating direction method of multipliers with indefinite proximal regularization

**DOI:** 10.1186/s13660-017-1295-1

**Published:** 2017-01-14

**Authors:** Min Sun, Jing Liu

**Affiliations:** 1 School of Management, Qufu Normal University, Shandong, 276826 P.R. China; 2 School of Mathematics and Statistics, Zaozhuang University, Shandong, 277160 P.R. China; 3 School of Data Sciences, Zhejiang University of Finance and Economics, Hangzhou, 310018 China

**Keywords:** 90C25, 90C30, proximal alternating direction method of multipliers, two-block separable convex minimization problem, compressive sensing

## Abstract

The proximal alternating direction method of multipliers (P-ADMM) is an efficient first-order method for solving the separable convex minimization problems. Recently, He *et al.* have further studied the P-ADMM and relaxed the proximal regularization matrix of its second subproblem to be indefinite. This is especially significant in practical applications since the indefinite proximal matrix can result in a larger step size for the corresponding subproblem and thus can often accelerate the overall convergence speed of the P-ADMM. In this paper, without the assumptions that the feasible set of the studied problem is bounded or the objective function’s component $\theta_{i}(\cdot)$ of the studied problem is strongly convex, we prove the worst-case $\mathcal{O}(1/t)$ convergence rate in an ergodic sense of the P-ADMM with a general Glowinski relaxation factor $\gamma\in(0,\frac{1+\sqrt{5}}{2})$, which is a supplement of the previously known results in this area. Furthermore, some numerical results on compressive sensing are reported to illustrate the effectiveness of the P-ADMM with indefinite proximal regularization.

## Introduction

Let $\theta_{i}:\mathcal{R}^{n_{i}}\rightarrow(-\infty, +\infty]\ (i=1,2)$ be two lower semicontinuous proper (not necessarily smooth) functions. This work aims to solve the following two-block separable convex minimization problem:

1 $$ \min \bigl\{ \theta_{1}(x_{1})+ \theta_{2}(x_{2})|A_{1}x_{1}+A_{2}x_{2}=b \bigr\} , $$

where $A_{i}\in\mathcal{R}^{l\times n_{i}}\ (i=1,2)$, $b\in\mathcal{R}^{l}$. If there are convex set constraints $x_{i}\in\mathcal{X}_{i}\ (i=1,2)$, where $\mathcal{X}_{i}\subseteq\mathcal{R}^{n_{i}}\ (i=1,2)$ are some simple convex set, such as the nonnegative cones or positive semi-definite cones, etc. Then, we can define the indicator function as $I_{\mathcal {X}_{i}}(\cdot)$ ($I_{\mathcal{X}_{i}}(x_{i})=0$ if $x_{i}\in\mathcal{X}_{i}$; otherwise, $I_{\mathcal{X}_{i}}(x_{i})=+\infty$), by which we can incorporate the constraints $x_{i}\in\mathcal{X}_{i}\ (i=1,2)$ into the objective function of (1), and get the following equivalent form:

$$\min \bigl\{ \theta_{1}(x_{1})+I_{\mathcal{X}_{1}}(x_{1})+ \theta _{2}(x_{2})+I_{\mathcal{X}_{2}}(x_{2})|A_{1}x_{1}+A_{2}x_{2}=b \bigr\} . $$

Then, we can further introduce some auxiliary variables and functions to rewrite the above problem as problem (1) (Please refer to [1] for more details). Therefore, problem (1) is quite general, and in fact problems like (1) come from diverse applications, such as the latent variable graphical model selection [2], the sparse inverse covariance selection [3], stable principal component pursuit with nonnegative constraint [4], and robust alignment for linearly correlated images [5], etc.

As one of the first-order methods, the following Algorithm 1, that is proximal alternating direction method of multipliers (P-ADMM) [6–8] is quite efficient for solving (1) or related problems, especially for large scale case.

**Algorithm 1:**
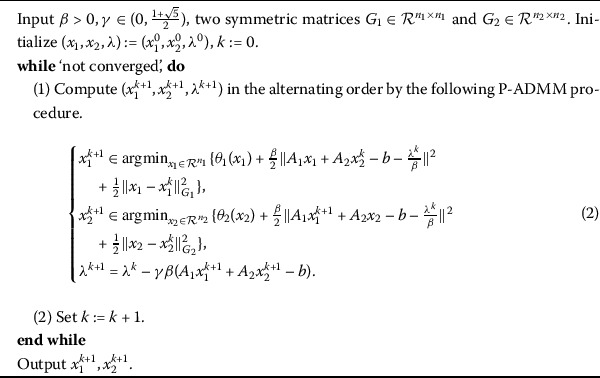
The P-ADMM for (1)

The parameter *γ* in the P-ADMM is called the Glowinski relaxation factor in the literature, and $\gamma>1$ can often accelerate the P-ADMM [9]. Due to its high efficiency, the P-ADMM has been intensively studied during the past few decades, and many scholars presented a lot of customized variants of the P-ADMM for some concrete separable minimization problems [10–12].

In this paper, we only focus our attention on the P-ADMM. In fact, the theory developed in this work can easily be extended to its various variants. Now, let us briefly analyze the structure advantages of the P-ADMM. Obviously, the P-ADMM fully utilizes the separable structure inherent to the original problem (1), which decouples the primal variable $(x_{1},x_{2})$ and get two subproblems with lower-dimension. Then, at each iteration, the computation of P-ADMM is dominated by solving its two subproblems. Fortunately, the two subproblems in (2) often admit closed-form solutions provided that $\theta_{i}(\cdot)\ (i=1,2)$ are some the functions (such as $\theta_{i}(\cdot)=\Vert \cdot \Vert _{1}$, $\Vert \cdot \Vert _{2}$ or $\Vert \cdot \Vert _{*}$) and the matrices $A_{i}\ (i=1,2)$ are unitary (*i.e.* $A_{i}^{\top}A_{i}\ (i=1,2)$ are the identity matrices). Even if $A_{i}\ (i=1,2)$ are not unitary, we can judiciously set $G_{i}=rI_{n_{i}}-\beta A_{i}^{\top}A_{i}$ with $r>\beta \Vert A_{i}^{\top}A_{i}\Vert \ (i=1,2)$, and then the two subproblems in the P-ADMM also have closed-form solutions in many practical applications. The global convergence of the P-ADMM with $\gamma=1$ has been proved in [10, 11] for some concrete models of (1), and in [13], Xu and Wu presented an elegant analysis of the global convergence of the P-ADMM with $\gamma\in(0,\frac{1+\sqrt{5}}{2})$ for the general model (1). Quite recently, He *et al.* [14] have further studied the P-ADMM and get some substantial advances by relaxing the matrix $G_{2}$ in the proximal regularization term of its second subproblem to be indefinite. This is quite preferred in practical applications since the indefinite proximal matrix can result in a larger step size for the subproblem and thus maybe accelerate the overall convergence speed of the P-ADMM.

Compared with the study of the global convergence of the P-ADMM, the research of its convergence rate is quite insubstantial in the literature. In [14, 15], under the assumption that the feasible set of (1) is bounded, He *et al.* have proved the worst-case $\mathcal{O}(1/t)$ convergence rate of the P-ADMM with $\gamma=1$, where *t* denotes the iteration counter. In [1], Lin *et al.* have presented a parallel version of the P-ADMM with the adaptive penalty *β*, and proved that the convergence rate of their new method is also $\mathcal{O}(1/t)$. In addition, Goldstein *et al.* [16] proved a better convergence rate than $\mathcal{O}(1/t)$ for the P-ADMM scheme with $\gamma=1$ and $G_{1}=0, G_{2}=0$ under the assumption that $\theta_{i}(\cdot)\ (i=1,2)$ are both strongly convex, which is usually violated in practice, and thus excludes many practical applications of the P-ADMM. Then, by introducing some free parameters $\alpha_{k}$ and $\gamma_{k}$, Xu [17] developed a new variant of the P-ADMM for (1), which refined the results in [16]. In fact, only under the assumption that the function $\theta_{2}(\cdot)$ is strongly convex, Xu [17] proved that the new method has $\mathcal{O}(1/t)$ convergence rate with constant parameters and enjoys $\mathcal {O}(1/t^{2})$ convergence rate with adaptive parameters.

In this paper, we aim to further improve the above results by removing the assumptions of the strong convexity of $\theta_{2}(\cdot)$ and the boundedness of the feasible set of (1), and prove that the P-ADMM for the convex minimization problem (1) has a worst-case $\mathcal{O}(1/t)$ convergence rate in an ergodic sense, which partially improves the results in [8, 13–15, 17].

The remaining of the paper is organized as follows. Section 2 gives some useful preliminaries. In Section 3, we prove the convergence rate of the P-ADMM in detail. In Section 4, a simple experiment on compressive sensing is conducted to demonstrate the effectiveness of the P-ADMM.

## Preliminaries

In this section, we summarize some basic concepts and preliminaries that will be used in the later discussion.

First, we list some notation to be used in this paper. $\langle\cdot ,\cdot\rangle$ denotes the inner product of $\mathcal{R}^{n}$; $G\succ0$ (or $G\succeq0$) denotes that the symmetric matrix *G* is positive definite (or positive semi-definite); If *G* is symmetric, we set $\Vert x\Vert _{G}^{2}=x^{\top}Gx$ though *G* maybe not positive definite. The effective domain of a function $f: \mathcal{X} \rightarrow(-\infty ,+\infty]$ is defined as $\operatorname{dom}(f):= \{x\in\mathcal{X}|f(x) < +\infty\}$. The set of all relative interior points of a given nonempty convex set $\mathcal{C}$ is denoted by $\operatorname{ri}(\mathcal{C})$.

A function $f:\mathcal{R}^{n}\rightarrow\mathcal{R}$ is convex iff

$$f \bigl(\alpha x+(1-\alpha)y \bigr)\leq\alpha f(x)+(1-\alpha)f(y), \quad\forall x,y\in \mathcal{R}^{n}, \alpha\in[0,1]. $$

Then, if $f:\mathcal{R}^{n}\rightarrow\mathcal{R}$ is convex, we have the following first-order necessary condition:

3 $$ f(x)\geq f(y)+\langle\xi, x-y\rangle,\quad \forall x,y\in \mathcal{R}^{n}, \xi\in\partial f(y), $$

where $\partial f(y)=\{\xi\in\mathcal{R}^{n}:f(\bar{y})\geq f(y)+\langle \xi,\bar{y}-y\rangle,\ \mathrm{for all}\ \bar{y}\in\mathcal{R}^{n}\}$ denotes the subdifferential of $f(\cdot)$ at the point *y*.

The following equality is used frequently in the paper:

4 $$ \langle x-y,x-z\rangle=\frac{1}{2} \bigl(\Vert x-y\Vert ^{2}+\Vert x-z\Vert ^{2}-\Vert y-z\Vert ^{2} \bigr),\quad \forall x,y,z\in\mathcal{R}^{n}. $$

From now on, we denote

$$x=(x_{1},x_{2}),\qquad \theta(x)=\theta_{1}(x_{1})+ \theta_{2}(x_{2}),\qquad A=(A_{1},A_{2}). $$

Throughout this paper, we make the following assumptions.

### Assumption 2.1

The functions $\theta_{i}(\cdot)\ (i=1,2)$ are both convex.

### Assumption 2.2

There is a point $(\hat{x}_{1},\hat{x}_{2})\in\operatorname {ri}(\operatorname{dom}(\theta_{1})\times\operatorname{dom}(\theta_{2}))$ such that $A_{1}\hat{x}_{1}+A_{2}\hat{x}_{2}=b$.

Then, under Assumption 2.2, it follows from Corollaries 28.2.2 and 28.3.1 in [18] that $({x}_{1}^{*},{x}_{2}^{*})\in\operatorname{ri}(\operatorname {dom}{(\theta_{1})}\times\operatorname{dom}{(\theta_{2})})$ is an optimal solution to problem (1) iff there exists a Lagrangian multiplier $\lambda^{*}\in\mathcal{R}^{l}$ such that $({x}_{1}^{*},{x}_{2}^{*},\lambda^{*})$ is a solution of the following KKT systems:

5 $$ \textstyle\begin{cases} 0\in\partial\theta_{1}(x_{1}^{*})-A_{1}^{\top}\lambda^{*},\\ 0\in\partial\theta_{2}(x_{2}^{*})-A_{2}^{\top}\lambda^{*},\\ A_{1}x_{1}^{*}+A_{2}x_{2}^{*}=b. \end{cases} $$

The set of the solutions of (5) is denoted by $\mathcal{W}^{*}$. By Assumption 2.1, (3), and (5), for any $({x}^{*},\lambda^{*})=({x}_{1}^{*},{x}_{2}^{*},\lambda^{*})\in\mathcal{W}^{*}$, we have the following useful inequality:

6 $$ \theta(x)-\theta \bigl(x^{*} \bigr)- \bigl\langle \lambda^{*}, Ax-b \bigr\rangle \geq0,\quad \forall x=(x_{1}, x_{2})\in \mathcal{R}^{n_{1}+n_{2}}. $$

### Assumption 2.3

The solution set $\mathcal{W}^{*}$ of the KKT systems (5) is nonempty, and at least one $({x}_{1}^{*},{x}_{2}^{*},\lambda^{*})\in\mathcal{W}^{*}$ with $\lambda^{*}\neq0$.

## Convergence rate of the P-ADMM

In this section, we aim to prove the convergence rate of the P-ADMM, and to accomplish this, we need to make some restrictions of the matrices $A_{i},G_{i}\ (i=1,2)$ included in the P-ADMM as follows.

### Assumption 3.1

(1) The matrix $G_{1}\succeq0$, and $A_{1}$ is full-column rank if $G_{1}=0$.

(2) The matrix $G_{2}$ is set as $G_{2}=\alpha\tau I_{n_{2}}-\beta A_{2}^{\top}A_{2}$ with $\tau>\beta \Vert A_{2}^{\top}A_{2}\Vert $, $\alpha\in(0,1]$, and $\alpha \geq(5-\min\{\gamma,1+\gamma-\gamma^{2}\})/5$.

### Remark 3.1

In [14], the parameter *α* can take any value of the interval $[0.8,1)$. Obviously, the parameter *α* in this paper can also obtain the lower bound 0.8 if $\gamma=1$.

Let us introduce some matrices to simplify our notation in the subsequent analysis. More specifically, we set

$$G= \begin{pmatrix}G_{1}&0\\0&G_{2} \end{pmatrix} $$

and

7 $$\begin{aligned} \begin{aligned} & \bar{G}_{2}=\tau I_{n_{2}}-\beta A_{2}^{\top}A_{2},\qquad M=G_{1}+\beta A_{1}^{\top}A_{1},\\ & N=G_{2}+\beta A_{2}^{\top}A_{2},\qquad H=G_{2}+\beta\min \bigl\{ \gamma,1+\gamma-\gamma^{2} \bigr\} A_{2}^{\top}A_{2}. \end{aligned} \end{aligned}$$

### Remark 3.2

From Assumption 3.1 and $\gamma\in(0,\frac{1+\sqrt {5}}{2})$, we see that the matrices $\bar{G}_{2}, M, N, H$ defined by (7) are all positive definite. However, the matrix $G_{2}$ defined in Assumption 3.1 may be indefinite. For example, when $\gamma=1, \alpha=0.8$, and $\tau=1.01\beta \Vert A_{2}^{\top}A_{2}\Vert $, then $G_{2}=-0.198\beta A_{2}^{\top}A_{2}$, which is obviously indefinite if the matrix $A_{2}$ is full-column rank.

### Remark 3.3

From the definitions of $G_{2}$ and $\bar{G}_{2}$, we have

8 $$ G_{2}=\alpha\bar{G}_{2}-(1-\alpha)\beta A_{2}^{\top}A_{2}. $$

Now, we start proving the convergence rate of the P-ADMM under Assumptions 2.1-2.3 and Assumption 3.1. Firstly, we prove three lemmas step by step.

### Lemma 3.1

*Let* $\{(x^{k},\lambda^{k})\}=\{(x_{1}^{k},x_{2}^{k},\lambda^{k})\} $ *be the sequence generated by the P*-*ADMM*. *Under Assumptions* 2.1-2.3, *for any* $(x_{1},x_{2},\lambda)\in\mathcal{R}^{n_{1}+n_{2}+l}$ *such that* $A_{1}x_{1}+A_{2}x_{2}=b$, *we have*

9 $$\begin{aligned} &\theta(x)-\theta\bigl(x^{k+1}\bigr) \\ &\quad\geq\frac{1}{2}\bigl(\bigl\Vert x^{k+1}-x\bigr\Vert _{G}^{2}-\bigl\Vert x^{k}-x\bigr\Vert _{G}^{2}+\bigl\Vert x^{k+1}-x^{k}\bigr\Vert _{G}^{2}\bigr)+\bigl\langle Ax^{k+1}-b,-{ \lambda}\bigr\rangle +\frac{2-\gamma }{2\beta\gamma^{2}}\bigl\Vert \lambda^{k+1}- \lambda^{k}\bigr\Vert ^{2} \\ &\qquad{}+\frac{1}{2\beta\gamma}\bigl(\bigl\Vert \lambda^{k+1}-\lambda \bigr\Vert ^{2}-\bigl\Vert \lambda^{k}-\lambda\bigr\Vert ^{2}\bigr)+\beta \bigl\langle Ax^{k+1}-b,A_{2}x_{2}^{k}-A_{2}x_{2}^{k+1} \bigr\rangle \\ &\qquad{}+\frac{\beta}{2}\bigl(\bigl\Vert A_{2}x_{2}^{k+1}-A_{2}x_{2} \bigr\Vert ^{2}-\bigl\Vert A_{2}x_{2}^{k}-A_{2}x_{2} \bigr\Vert ^{2}+\bigl\Vert A_{2}x_{2}^{k+1}-A_{2}x_{2}^{k} \bigr\Vert ^{2}\bigr). \end{aligned}$$

### Proof

Note that the optimality condition for the first subproblem (*i.e.*, the subproblem with respect to $x_{1}$) in (2) is

10 $$\begin{aligned} 0&=\nabla\theta_{1}\bigl(x_{1}^{k+1} \bigr)+\beta A_{1}^{\top}\biggl(A_{1}x_{1}^{k+1}+A_{2}x_{2}^{k}-b- \frac{1}{\beta}\lambda ^{k}\biggr)+G_{1} \bigl(x_{1}^{k+1}-x_{1}^{k}\bigr) \\ & =\nabla\theta_{1}\bigl(x_{1}^{k+1} \bigr)-A_{1}^{\top}\tilde{\lambda}^{k}+\beta A_{1}^{\top}A_{2}\bigl(x_{2}^{k}-x_{2}^{k+1} \bigr)+G_{1}\bigl(x_{1}^{k+1}-x_{1}^{k} \bigr), \end{aligned}$$

where $\nabla\theta_{1}(x_{1}^{k+1})$ is a subgradient of $\theta_{1}(\cdot)$ at $x_{1}^{k+1}$, $\tilde{\lambda}^{k}=\lambda^{k}-\beta (A_{1}x_{1}^{k+1}+A_{2}x_{2}^{k+1}-b)$, and the second equality uses the updating formula for *λ* in (2). Then (10) can be rewritten as

11 $$\begin{aligned} 0={}&\bigl\langle x_{1}^{k+1}-x_{1}, \nabla\theta_{1}\bigl(x_{1}^{k+1}\bigr)\bigr\rangle +\bigl\langle x_{1}^{k+1}-x_{1}, G_{1}\bigl(x_{1}^{k+1}-x_{1}^{k} \bigr)+\beta A_{1}^{\top}A_{2}\bigl(x_{2}^{k}-x_{2}^{k+1} \bigr)\bigr\rangle \\ &{} +\bigl\langle A_{1}\bigl(x_{1}^{k+1}-x_{1} \bigr),-\tilde{\lambda}^{k}\bigr\rangle \\ \geq{}&\theta_{1}\bigl(x_{1}^{k+1}\bigr)- \theta_{1}(x_{1})+\bigl\langle x_{1}^{k+1}-x_{1}, G_{1}\bigl(x_{1}^{k+1}-x_{1}^{k} \bigr)+\beta A_{1}^{\top}A_{2}\bigl(x_{2}^{k}-x_{2}^{k+1} \bigr)\bigr\rangle \\ &{} +\bigl\langle A_{1}\bigl(x_{1}^{k+1}-x_{1} \bigr),-\tilde{\lambda}^{k}\bigr\rangle , \end{aligned}$$

where the inequality comes from the convexity of $\theta_{1}(\cdot)$ and (3). Similarly, the optimality condition for the second subproblem (*i.e.*, the subproblem with respect to $x_{2}$) in (2) gives

$$\begin{aligned} 0&=\nabla\theta_{2} \bigl(x_{2}^{k+1} \bigr)+\beta A_{2}^{\top}\biggl(A_{1}x_{1}^{k+1}+A_{2}x_{2}^{k+1}-b- \frac{1}{\beta}\lambda ^{k} \biggr)+G_{2} \bigl(x_{2}^{k+1}-x_{2}^{k} \bigr) \\ & =\nabla\theta_{1} \bigl(x_{1}^{k+1} \bigr)-A_{2}^{\top}\tilde{\lambda }^{k}+G_{2} \bigl(x_{2}^{k+1}-x_{2}^{k} \bigr){,} \end{aligned}$$

*i.e.*,

12 $$\begin{aligned} 0={}&\bigl\langle x_{2}^{k+1}-x_{2}, \nabla\theta_{2}\bigl(x_{2}^{k+1}\bigr)\bigr\rangle + \bigl\langle A_{2}\bigl(x_{2}^{k+1}-x_{2} \bigr),-\tilde{\lambda}^{k}\bigr\rangle +\bigl\langle x_{2}^{k+1}-x_{2}, G_{2} \bigl(x_{2}^{k+1}-x_{2}^{k}\bigr)\bigr\rangle \\ \geq{}&\theta_{2}\bigl(x_{2}^{k+1}\bigr)- \theta_{2}(x_{2})+\bigl\langle x_{2}^{k+1}-x_{2}, G_{2}\bigl(x_{2}^{k+1}-x_{2}^{k} \bigr)\bigr\rangle +\bigl\langle A_{2}\bigl(x_{2}^{k+1}-x_{2} \bigr),-\tilde{\lambda }^{k}\bigr\rangle , \end{aligned}$$

where the inequality follows from the convexity of $\theta_{2}(\cdot)$ and (3). Then, adding (11) and (12), we obtain

13 $$\begin{aligned} &\theta(x)-\theta\bigl(x^{k+1}\bigr) \\ &\quad\geq\sum_{i=1}^{2}\bigl\langle x_{i}^{k+1}-x_{i}, G_{i} \bigl(x_{i}^{k+1}-x_{i}^{k}\bigr)\bigr\rangle +\sum_{i=1}^{2}\bigl\langle A_{i}\bigl(x_{i}^{k+1}-x_{i}\bigr),- \tilde{\lambda}^{k}\bigr\rangle \\ &\qquad{}+\beta\bigl\langle A_{1}x_{1}^{k+1}-A_{1}x_{1},A_{2}x_{2}^{k}-A_{2}x_{2}^{k+1} \bigr\rangle \\ &\quad=\sum_{i=1}^{2}\bigl\langle x_{i}^{k+1}-x_{i}, G_{i} \bigl(x_{i}^{k+1}-x_{i}^{k}\bigr)\bigr\rangle +\bigl\langle Ax^{k+1}-b,-\tilde{\lambda }^{k}\bigr\rangle +\beta\bigl\langle A_{1}x_{1}^{k+1}-A_{1}x_{1},A_{2}x_{2}^{k}-A_{2}x_{2}^{k+1} \bigr\rangle \\ &\quad=\sum_{i=1}^{2}\bigl\langle x_{i}^{k+1}-x_{i}, G_{i} \bigl(x_{i}^{k+1}-x_{i}^{k}\bigr)\bigr\rangle +\bigl\langle Ax^{k+1}-b,-\tilde{\lambda }^{k}\bigr\rangle \\ &\qquad{}+\beta\bigl\langle A_{1}x_{1}^{k+1}+A_{2}x_{2}^{k+1}-A_{1}x_{1}-A_{2}x_{2},A_{2}x_{2}^{k}-A_{2}x_{2}^{k+1} \bigr\rangle \\ &\qquad{}+\beta\bigl\langle A_{2}x_{2}-A_{2}x_{2}^{k+1},A_{2}x_{2}^{k}-A_{2}x_{2}^{k+1} \bigr\rangle \\ &\quad=\sum_{i=1}^{2}\bigl\langle x_{i}^{k+1}-x_{i}, G_{i} \bigl(x_{i}^{k+1}-x_{i}^{k}\bigr)\bigr\rangle +\bigl\langle Ax^{k+1}-b,-\tilde{\lambda }^{k}\bigr\rangle \\ &\qquad{}+\beta\bigl\langle Ax^{k+1}-b,A_{2}x_{2}^{k}-A_{2}x_{2}^{k+1} \bigr\rangle +\beta\bigl\langle A_{2}x_{2}-A_{2}x_{2}^{k+1},A_{2}x_{2}^{k}-A_{2}x_{2}^{k+1} \bigr\rangle \\ &\quad=\frac{1}{2}\sum_{i=1}^{2} \bigl(\bigl\Vert x_{i}^{k+1}-x_{i}\bigr\Vert _{G_{i}}^{2}-\bigl\Vert x_{i}^{k}-x_{i} \bigr\Vert _{G_{i}}^{2}+\bigl\Vert x_{i}^{k+1}-x_{i}^{k} \bigr\Vert _{G_{i}}^{2}\bigr)+\bigl\langle Ax^{k+1}-b,- \tilde{\lambda}^{k}\bigr\rangle \\ &\qquad{}+\beta\bigl\langle Ax^{k+1}-b,A_{2}x_{2}^{k}-A_{2}x_{2}^{k+1} \bigr\rangle \\ &\qquad{}+\frac{\beta}{2}\bigl(\bigl\Vert A_{2}x_{2}^{k+1}-A_{2}x_{2} \bigr\Vert ^{2}-\bigl\Vert A_{2}x_{2}^{k}-A_{2}x_{2} \bigr\Vert ^{2}+\bigl\Vert A_{2}x_{2}^{k+1}-A_{2}x_{2}^{k} \bigr\Vert ^{2}\bigr), \end{aligned}$$

where the last equality comes from the identity (4). Now, let us deal with the term $\langle Ax^{k+1}-b,-\tilde{\lambda }^{k}\rangle$ on the right side of (13). Specifically, from the updating formula for *λ* in (2) again, we can get

14 $$\begin{aligned} &\bigl\langle Ax^{k+1}-b,-\tilde{ \lambda}^{k}\bigr\rangle \\ &\quad=\bigl\langle Ax^{k+1}-b,-\lambda\bigr\rangle +\frac{1}{\beta} \bigl\langle \lambda^{k}-\tilde{\lambda}^{k},\lambda-\tilde{ \lambda}^{k}\bigr\rangle \\ &\quad=\bigl\langle Ax^{k+1}-b,-\lambda\bigr\rangle +\frac{1}{\beta} \biggl\langle \lambda^{k}-\frac{1}{\gamma}{\lambda}^{k+1}+ \frac{1-\gamma}{\gamma}\lambda ^{k},\lambda-\frac{1}{\gamma}{ \lambda}^{k+1}+\frac{1-\gamma}{\gamma }\lambda^{k}\biggr\rangle \\ &\quad= \bigl\langle Ax^{k+1}-b,-\lambda\bigr\rangle + \frac{1}{\beta}\biggl\langle \frac{1}{\gamma}\lambda^{k}- \frac{1}{\gamma}{\lambda}^{k+1},\lambda -\lambda^{k}+ \frac{1}{\gamma}\lambda^{k}-\frac{1}{\gamma}{\lambda }^{k+1}\biggr\rangle \\ &\quad =\bigl\langle Ax^{k+1}-b,-\lambda\bigr\rangle +\frac{1}{\beta\gamma } \bigl\langle \lambda^{k}-{\lambda}^{k+1},\lambda- \lambda^{k}\bigr\rangle +\frac {1}{\beta\gamma^{2}}\bigl\Vert \lambda^{k+1}-\lambda^{k}\bigr\Vert ^{2} \\ &\quad=\bigl\langle Ax^{k+1}-b,-\lambda\bigr\rangle +\frac{1}{2\beta\gamma } \bigl(\bigl\Vert \lambda^{k+1}-\lambda\bigr\Vert ^{2}-\bigl\Vert \lambda^{k}-\lambda\bigr\Vert ^{2}\bigr)+ \frac{2-\gamma }{2\beta\gamma^{2}}\bigl\Vert \lambda^{k+1}-\lambda^{k}\bigr\Vert ^{2}, \end{aligned}$$

where the second equality comes from $\lambda^{k+1}=\lambda^{k}-\gamma (\lambda^{k}-\tilde{\lambda}^{k})$, and the last equality uses the identity (4). Then, substituting (14) into (13) yields (9). This completes the proof. □

The following lemma aims to further refine the crossing term $\beta \langle Ax^{k+1}-b,A_{2}x_{2}^{k}-A_{2}x_{2}^{k+1}\rangle$ on the right side of (9).

### Lemma 3.2

*Let* $\{(x^{k},\lambda^{k})\}=\{(x_{1}^{k},x_{2}^{k},\lambda^{k})\} $ *be the sequence generated by the P*-*ADMM*. *Under Assumptions* 2.1-2.3, *for any* $(x_{1},x_{2},\lambda)\in\mathcal{R}^{n_{1}+n_{2}+l}$ *such that* $A_{1}x_{1}+A_{2}x_{2}=b$, *we have*

15 $$\begin{aligned} &\theta(x)-\theta\bigl(x^{k+1}\bigr) \\ &\quad\geq\frac{1}{2}\bigl(\bigl\Vert x^{k+1}-x\bigr\Vert _{G}^{2}-\bigl\Vert x^{k}-x\bigr\Vert _{G}^{2}+\bigl\Vert x^{k+1}-x^{k}\bigr\Vert _{G}^{2}\bigr)+\bigl\langle Ax^{k+1}-b,-{ \lambda}\bigr\rangle \\ &\qquad{}+\frac{1}{2\beta\gamma}\bigl(\bigl\Vert \lambda^{k+1}-\lambda \bigr\Vert ^{2}-\bigl\Vert \lambda^{k}-\lambda\bigr\Vert ^{2}\bigr)+\frac{2-\gamma}{2\beta\gamma^{2}}\bigl\Vert \lambda ^{k+1}-\lambda^{k}\bigr\Vert ^{2} \\ &\qquad{}+(1-\gamma)\beta\bigl\langle A_{2}\bigl(x_{2}^{k}-x_{2}^{k+1} \bigr),A_{1}x_{1}^{k}+A_{2}x_{2}^{k}-b \bigr\rangle +\bigl\Vert x_{2}^{k+1}-x_{2}^{k} \bigr\Vert _{G_{2}}^{2} \\ &\qquad{}+\bigl\langle x_{2}^{k}-x_{2}^{k+1}, G_{2}\bigl(x_{2}^{k}-x_{2}^{k-1} \bigr)\bigr\rangle \\ &\qquad{}+\frac{\beta}{2}\bigl(\bigl\Vert A_{2}x_{2}^{k+1}-A_{2}x_{2} \bigr\Vert ^{2}-\bigl\Vert A_{2}x_{2}^{k}-A_{2}x_{2} \bigr\Vert ^{2}+\bigl\Vert A_{2}x_{2}^{k+1}-A_{2}x_{2}^{k} \bigr\Vert ^{2}\bigr). \end{aligned}$$

### Proof

Setting $x_{2}=x_{2}^{k}$ in (12), we get

$$0\geq\theta_{2} \bigl(x_{2}^{k+1} \bigr)- \theta_{2} \bigl(x_{2}^{k} \bigr)+ \bigl\langle x_{2}^{k+1}-x_{2}^{k}, G_{2} \bigl(x_{2}^{k+1}-x_{2}^{k} \bigr) \bigr\rangle + \bigl\langle A_{2} \bigl(x_{2}^{k+1}-x_{2}^{k} \bigr),-\tilde {\lambda}^{k} \bigr\rangle . $$

That is,

16 $$ \theta_{2} \bigl(x_{2}^{k} \bigr)- \theta_{2} \bigl(x_{2}^{k+1} \bigr)- \bigl\langle A_{2} \bigl(x_{2}^{k+1}-x_{2}^{k} \bigr),-\tilde{\lambda}^{k} \bigr\rangle \geq \bigl\Vert x_{2}^{k+1}-x_{2}^{k} \bigr\Vert _{G_{2}}^{2}. $$

Similarly, taking $x_{2}=x_{2}^{k+1}$ in (12) for $k:=k-1$, and thus we have

$$0\geq\theta_{2} \bigl(x_{2}^{k} \bigr)- \theta_{2} \bigl(x_{2}^{k+1} \bigr)+ \bigl\langle x_{2}^{k}-x_{2}^{k+1}, G_{2} \bigl(x_{2}^{k}-x_{2}^{k-1} \bigr) \bigr\rangle + \bigl\langle A_{2} \bigl(x_{2}^{k}-x_{2}^{k+1} \bigr),-\tilde {\lambda}^{k-1} \bigr\rangle . $$

That is,

17 $$ \theta_{2} \bigl(x_{2}^{k+1} \bigr)- \theta _{2} \bigl(x_{2}^{k} \bigr)- \bigl\langle A_{2} \bigl(x_{2}^{k}-x_{2}^{k+1} \bigr),-\tilde{\lambda}^{k-1} \bigr\rangle \geq \bigl\langle x_{2}^{k}-x_{2}^{k+1}, G_{2} \bigl(x_{2}^{k}-x_{2}^{k+1} \bigr) \bigr\rangle . $$

Adding (16) and (17), we obtain

18 $$ \bigl\langle A_{2} \bigl(x_{2}^{k}-x_{2}^{k+1} \bigr),\tilde {\lambda}^{k-1}-\tilde{\lambda}^{k} \bigr\rangle \geq \bigl\Vert x_{2}^{k+1}-x_{2}^{k} \bigr\Vert _{G_{2}}^{2}+ \bigl\langle x_{2}^{k}-x_{2}^{k+1}, G_{2} \bigl(x_{2}^{k}-x_{2}^{k-1} \bigr) \bigr\rangle . $$

We have

$$\begin{aligned} &\tilde{\lambda}^{k-1}-\tilde{\lambda}^{k} \\ &\quad=\tilde{\lambda}^{k-1}- \bigl[\lambda^{k}-\beta \bigl(A_{1}x_{1}^{k+1}+A_{2}x_{2}^{k+1}-b \bigr) \bigr] \\ &\quad=\tilde{\lambda}^{k-1}- \bigl[\lambda^{k-1}-\gamma\beta \bigl(A_{1}x_{1}^{k}+A_{2}x_{2}^{k}-b \bigr)-\beta \bigl(A_{1}x_{1}^{k+1}+A_{2}x_{2}^{k+1}-b \bigr) \bigr] \\ &\quad =-(1-\gamma)\beta \bigl(A_{1}x_{1}^{k}+A_{2}x_{2}^{k}-b \bigr)+\beta \bigl(A_{1}x_{1}^{k+1}+A_{2}x_{2}^{k+1}-b \bigr). \end{aligned}$$

Substituting the above equality into (18), we obtain

19 $$\begin{aligned} &\beta\bigl\langle A_{2} \bigl(x_{2}^{k}-x_{2}^{k+1} \bigr),A_{1}x_{1}^{k+1}+A_{2}x_{2}^{k+1}-b \bigr\rangle \\ &\quad\geq(1-\gamma)\beta\bigl\langle A_{2}\bigl(x_{2}^{k}-x_{2}^{k+1} \bigr),A_{1}x_{1}^{k}+A_{2}x_{2}^{k}-b \bigr\rangle +\bigl\Vert x_{2}^{k+1}-x_{2}^{k} \bigr\Vert _{G_{2}}^{2} \\ &\qquad{}+\bigl\langle x_{2}^{k}-x_{2}^{k+1}, G_{2}\bigl(x_{2}^{k}-x_{2}^{k-1} \bigr)\bigr\rangle . \end{aligned}$$

Then, substituting (19) into (9) yields (15). The proof is completed.

Now, let us deal with the term $\frac{2-\gamma}{2\beta\gamma^{2}}\Vert \lambda ^{k+1}-\lambda^{k}\Vert ^{2} +(1-\gamma)\beta\langle A_{2}(x_{2}^{k}-x_{2}^{k+1}),A_{1}x_{1}^{k}+A_{2}x_{2}^{k}-b\rangle+\frac{\beta}{2}\Vert A_{2}x_{2}^{k+1}-A_{2}x_{2}^{k}\Vert ^{2}$ on the right side of (15). □

### Lemma 3.3

*Let* $\{(x^{k},\lambda^{k})\}=\{(x_{1}^{k},x_{2}^{k},\lambda^{k})\} $ *be the sequence generated by the P*-*ADMM*. *Then*, *we have*

20 $$\begin{aligned} &\frac{2-\gamma}{2\beta\gamma^{2}}\bigl\Vert \lambda^{k+1}- \lambda ^{k}\bigr\Vert ^{2}+(1-\gamma)\beta\bigl\langle A_{2}\bigl(x_{2}^{k}-x_{2}^{k+1} \bigr),A_{1}x_{1}^{k}+A_{2}x_{2}^{k}-b \bigr\rangle +\frac{\beta}{2}\bigl\Vert A_{2}x_{2}^{k+1}-A_{2}x_{2}^{k} \bigr\Vert ^{2} \\ &\quad\geq\frac{1}{2\beta\gamma^{2}}\max\bigl\{ 1-\gamma,1-\gamma^{-1}\bigr\} \bigl(\bigl\Vert \lambda^{k+1}-\lambda^{k}\bigr\Vert ^{2}-\bigl\Vert \lambda^{k}-\lambda^{k-1}\bigr\Vert ^{2}\bigr) \\ &\qquad{}+\frac{\beta\varrho}{2}\biggl(\frac{1}{\gamma^{3}\beta^{2}}\bigl\Vert \lambda^{k+1}-\lambda^{k}\bigr\Vert ^{2}+\bigl\Vert A_{2}\bigl(x_{2}^{k}-x_{2}^{k+1} \bigr)\bigr\Vert ^{2}\biggr), \end{aligned}$$

*where* $\varrho=\min\{\gamma,1+\gamma-\gamma^{2}\}$.

### Proof

Obviously, by the updating formula for *λ* in (2), we have

21 $$ \bigl\langle A_{2} \bigl(x_{2}^{k}-x_{2}^{k+1} \bigr),A_{1}x_{1}^{k}+A_{2}x_{2}^{k}-b \bigr\rangle =\frac{1}{\beta \gamma} \bigl\langle A_{2} \bigl(x_{2}^{k}-x_{2}^{k+1} \bigr), \lambda^{k-1}-\lambda^{k} \bigr\rangle . $$

Then, applying the Cauchy-Schwartz inequality, we can get

$$\textstyle\begin{cases} (1-\gamma)\beta\langle A_{2}(x_{2}^{k}-x_{2}^{k+1}),(\lambda^{k-1}-\lambda ^{k})/(\beta\gamma)\rangle\\ \quad\geq-\frac{(1-\gamma)\beta}{2}(\Vert A_{2}(x_{2}^{k}-x_{2}^{k+1})\Vert ^{2}+\frac{1}{\gamma^{2}\beta^{2}}\Vert \lambda ^{k-1}-\lambda^{k}\Vert ^{2})\\ \qquad \mathrm{if\ } \gamma\in(0,1],\\ (1-\gamma)\beta\langle A_{2}(x_{2}^{k}-x_{2}^{k+1}),(\lambda^{k-1}-\lambda ^{k})/(\beta\gamma)\rangle\\ \quad\geq-\frac{(\gamma-1)\beta}{2}(\gamma \Vert A_{2}(x_{2}^{k}-x_{2}^{k+1})\Vert ^{2}+\frac{1}{\gamma^{3}\beta^{2}}\Vert \lambda ^{k-1}-\lambda^{k}\Vert ^{2})\\ \qquad\mathrm{if\ } \gamma\in(1,+\infty). \end{cases} $$

Then, substituting the above two inequalities into (21), and by some simple manipulations, we obtain

$$\begin{aligned} &\frac{2-\gamma}{2\beta\gamma^{2}} \bigl\Vert \lambda^{k+1}-\lambda^{k} \bigr\Vert ^{2}+(1-\gamma )\beta \bigl\langle A_{2} \bigl(x_{2}^{k}-x_{2}^{k+1} \bigr),A_{1}x_{1}^{k}+A_{2}x_{2}^{k}-b \bigr\rangle +\frac{\beta}{2} \bigl\Vert A_{2}x_{2}^{k+1}-A_{2}x_{2}^{k} \bigr\Vert ^{2} \\ &\quad\geq\frac{1}{2\beta\gamma^{2}}\max \bigl\{ 1-\gamma,1-\gamma^{-1} \bigr\} \bigl( \bigl\Vert \lambda ^{k+1}-\lambda^{k} \bigr\Vert ^{2}- \bigl\Vert \lambda^{k}-\lambda^{k-1} \bigr\Vert ^{2} \bigr) \\ &\qquad{}+\frac{\beta}{2}\min \bigl\{ \gamma,1+\gamma-\gamma^{2} \bigr\} \biggl(\frac{1}{\gamma ^{3}\beta^{2}} \bigl\Vert \lambda^{k+1}- \lambda^{k} \bigr\Vert ^{2}+ \bigl\Vert A_{2} \bigl(x_{2}^{k}-x_{2}^{k+1} \bigr) \bigr\Vert ^{2} \biggr), \end{aligned}$$

which is the same as the assertion (20), and the lemma is thus proved.

Substituting (20) into (15), we get the following important inequality:

22 $$\begin{aligned} &\theta(x)-\theta\bigl(x^{k+1}\bigr) \\ &\quad\geq\frac{1}{2}\bigl(\bigl\Vert x^{k+1}-x\bigr\Vert _{G}^{2}-\bigl\Vert x^{k}-x\bigr\Vert _{G}^{2}+\bigl\Vert x^{k+1}-x^{k}\bigr\Vert _{G}^{2}\bigr)+\bigl\langle Ax^{k+1}-b,-{ \lambda}\bigr\rangle \\ &\qquad{}+\frac{1}{2\beta\gamma}\bigl(\bigl\Vert \lambda^{k+1}-\lambda \bigr\Vert ^{2}-\bigl\Vert \lambda^{k}-\lambda\bigr\Vert ^{2}\bigr) \\ &\qquad{}+\frac{1}{2\beta\gamma^{2}}\max\bigl\{ 1-\gamma,1-\gamma ^{-1}\bigr\} \bigl(\bigl\Vert \lambda^{k+1}- \lambda^{k}\bigr\Vert ^{2}-\bigl\Vert \lambda^{k}-\lambda^{k-1}\bigr\Vert ^{2}\bigr) \\ &\qquad{}+\bigl\Vert x_{2}^{k+1}-x_{2}^{k} \bigr\Vert _{G_{2}}^{2}+\bigl\langle x_{2}^{k}-x_{2}^{k+1}, G_{2}\bigl(x_{2}^{k}-x_{2}^{k-1} \bigr)\bigr\rangle \\ &\qquad{}+\frac{\beta}{2}\bigl(\bigl\Vert A_{2}x_{2}^{k+1}-A_{2}x_{2} \bigr\Vert ^{2}-\bigl\Vert A_{2}x_{2}^{k}-A_{2}x_{2} \bigr\Vert ^{2}\bigr) \\ &\qquad{}+\frac{\beta}{2}\min\bigl\{ \gamma,1+\gamma-\gamma^{2}\bigr\} \biggl(\frac {1}{\gamma^{3}\beta^{2}}\bigl\Vert \lambda^{k+1}- \lambda^{k}\bigr\Vert ^{2}+\bigl\Vert A_{2} \bigl(x_{2}^{k}-x_{2}^{k+1}\bigr)\bigr\Vert ^{2}\biggr). \end{aligned}$$

Now, let us deal with all the terms related with the variable $x_{2}$ on the right side of (22). From the definition of the matrices $G_{2}, N$ and (8), we have

$$\begin{aligned} &\frac{1}{2} \bigl( \bigl\Vert x_{2}^{k+1}-x_{2} \bigr\Vert _{G_{2}+\beta A_{2}^{\top}A_{2}}^{2}- \bigl\Vert x_{2}^{k}-x_{2} \bigr\Vert _{G_{2}+\beta A_{2}^{\top}A_{2}}^{2} \bigr)\\ &\qquad{}+\frac{1}{2} \bigl\Vert x_{2}^{k+1}-x_{2}^{k} \bigr\Vert _{G_{2}+\beta\varrho A_{2}^{\top}A_{2}}^{2} \\ &\qquad{}+ \bigl\Vert x_{2}^{k+1}-x_{2}^{k} \bigr\Vert _{G_{2}}^{2}+ \bigl\langle x_{2}^{k}-x_{2}^{k+1}, G_{2} \bigl(x_{2}^{k}-x_{2}^{k-1} \bigr) \bigr\rangle \\ &\quad=\frac{1}{2} \bigl( \bigl\Vert x_{2}^{k+1}-x_{2} \bigr\Vert _{N}^{2}- \bigl\Vert x_{2}^{k}-x_{2} \bigr\Vert _{N}^{2} \bigr)+\frac {1}{2} \bigl\Vert x_{2}^{k+1}-x_{2}^{k} \bigr\Vert _{3\alpha\bar{G}_{2}-(3-3\alpha-\varrho)\beta A_{2}^{\top}A_{2}}^{2} \\ &\qquad{}+\alpha \bigl(x_{2}^{k}-x_{2}^{k+1} \bigr)^{\top}\bar{G}_{2} \bigl(x_{2}^{k}-x_{2}^{k-1} \bigr)-(1-\alpha )\beta \bigl(x_{2}^{k}-x_{2}^{k+1} \bigr)^{\top}\bigl(A_{2}^{\top}A_{2} \bigr) \bigl(x_{2}^{k}-x_{2}^{k-1} \bigr) \\ &\quad\geq\frac{1}{2} \bigl( \bigl\Vert x_{2}^{k+1}-x_{2} \bigr\Vert _{N}^{2}- \bigl\Vert x_{2}^{k}-x_{2} \bigr\Vert _{N}^{2} \bigr)+\frac {1}{2} \bigl\Vert x_{2}^{k+1}-x_{2}^{k} \bigr\Vert _{3\alpha\bar{G}_{2}-(3-3\alpha-\varrho)\beta A_{2}^{\top}A_{2}}^{2} \\ &\qquad{}-\frac{\alpha}{2} \bigl( \bigl\Vert x_{2}^{k}-x_{2}^{k+1} \bigr\Vert _{\bar{G}_{2}}^{2}+ \bigl\Vert x_{2}^{k}-x_{2}^{k-1} \bigr\Vert _{\bar{G}_{2}}^{2} \bigr)\\ &\qquad{}-\frac{(1-\alpha)\beta}{2} \bigl( \bigl\Vert A_{2} \bigl(x_{2}^{k}-x_{2}^{k+1} \bigr) \bigr\Vert ^{2}+ \bigl\Vert A_{2} \bigl(x_{2}^{k}-x_{2}^{k-1} \bigr) \bigr\Vert ^{2} \bigr) \\ &\quad=\frac{1}{2} \bigl( \bigl\Vert x_{2}^{k+1}-x_{2} \bigr\Vert _{N}^{2}- \bigl\Vert x_{2}^{k}-x_{2} \bigr\Vert _{N}^{2} \bigr)+\frac {\alpha}{2} \bigl\Vert x_{2}^{k+1}-x_{2}^{k} \bigr\Vert _{\bar{G}_{2}}^{2}\\ &\qquad{}+\frac{\alpha}{2} \bigl( \bigl\Vert x_{2}^{k+1}-x_{2}^{k} \bigr\Vert _{\bar{G}_{2}}^{2}- \bigl\Vert x_{2}^{k}-x_{2}^{k-1} \bigr\Vert _{\bar{G}_{2}}^{2} \bigr) \\ &\qquad{}+\frac{(1-\alpha)\beta}{2} \bigl( \bigl\Vert A_{2} \bigl(x_{2}^{k}-x_{2}^{k+1} \bigr) \bigr\Vert ^{2}- \bigl\Vert A_{2} \bigl(x_{2}^{k}-x_{2}^{k-1} \bigr) \bigr\Vert ^{2} \bigr)\\ &\qquad{}+\frac{\beta(\varrho-5+5\alpha)}{2} \bigl\Vert A_{2} \bigl(x_{2}^{k}-x_{2}^{k+1} \bigr) \bigr\Vert ^{2}. \end{aligned}$$

Then, substituting the above inequality into (22), we can obtain

23 $$\begin{aligned} &\theta(x)-\theta\bigl(x^{k+1}\bigr) \\ &\quad\geq\bigl\langle Ax^{k+1}-b,-{\lambda}\bigr\rangle + \frac{1}{2}\bigl(\bigl\Vert x_{1}^{k+1}-x_{1} \bigr\Vert _{G_{1}}^{2}-\bigl\Vert x_{1}^{k}-x_{1} \bigr\Vert _{G_{1}}^{2}+\bigl\Vert x_{1}^{k+1}-x_{1}^{k} \bigr\Vert _{G_{1}}^{2}\bigr) \\ &\qquad{}+\frac{1}{2}\bigl(\bigl\Vert x_{2}^{k+1}-x_{2} \bigr\Vert _{N}^{2}-\bigl\Vert x_{2}^{k}-x_{2} \bigr\Vert _{N}^{2}\bigr) \\ &\qquad{}+\frac{1}{2}\bigl(\bigl\Vert x_{2}^{k}-x_{2}^{k+1}\bigr\Vert _{\alpha\bar{G}_{2}+\beta(1-\alpha )A_{2}^{\top}A_{2}}^{2}-\bigl\Vert x_{2}^{k-1}-x_{2}^{k} \bigr\Vert _{\alpha\bar{G}_{2}+\beta(1-\alpha )A_{2}^{\top}A_{2}}^{2}\bigr) \\ &\qquad{}+\frac{1}{2\beta\gamma}\bigl(\bigl\Vert \lambda^{k+1}-\lambda \bigr\Vert ^{2}-\bigl\Vert \lambda^{k}-\lambda\bigr\Vert ^{2}\bigr) \\ &\qquad{}+\frac{1}{2\beta\gamma^{2}}\max\bigl\{ 1-\gamma,1-\gamma ^{-1}\bigr\} \bigl(\bigl\Vert \lambda^{k+1}- \lambda^{k}\bigr\Vert ^{2}-\bigl\Vert \lambda^{k}-\lambda^{k-1}\bigr\Vert ^{2}\bigr) \\ &\qquad{}+\frac{\alpha}{2}\bigl\Vert x_{2}^{k+1}-x_{2}^{k} \bigr\Vert _{\bar{G}_{2}}^{2}+\frac {\beta(\varrho-5+5\alpha)}{2}\bigl\Vert A_{2}\bigl(x_{2}^{k}-x_{2}^{k+1} \bigr)\bigr\Vert ^{2}+\frac{\varrho }{2\gamma^{3}\beta}\bigl\Vert \lambda^{k+1}-\lambda^{k}\bigr\Vert ^{2} \\ &\quad\geq\bigl\langle Ax^{k+1}-b,-{\lambda}\bigr\rangle + \frac{1}{2}\bigl(\bigl\Vert x_{1}^{k+1}-x_{1} \bigr\Vert _{G_{1}}^{2}-\bigl\Vert x_{1}^{k}-x_{1} \bigr\Vert _{G_{1}}^{2}\bigr)+\frac{1}{2}\bigl(\bigl\Vert x_{2}^{k+1}-x_{2}\bigr\Vert _{N}^{2}-\bigl\Vert x_{2}^{k}-x_{2} \bigr\Vert _{N}^{2}\bigr) \\ &\qquad{}+\frac{1}{2}\bigl(\bigl\Vert x_{2}^{k}-x_{2}^{k+1} \bigr\Vert _{\alpha\bar{G}_{2}+\beta (1-\alpha)A_{2}^{\top}A_{2}}^{2}-\bigl\Vert x_{2}^{k-1}-x_{2}^{k} \bigr\Vert _{\alpha\bar{G}_{2}+\beta (1-\alpha)A_{2}^{\top}A_{2}}^{2}\bigr) \\ &\qquad{}+\frac{1}{2\beta\gamma}\bigl(\bigl\Vert \lambda^{k+1}-\lambda \bigr\Vert ^{2}-\bigl\Vert \lambda^{k}-\lambda\bigr\Vert ^{2}\bigr) \\ &\qquad{}+\frac{1}{2\beta\gamma^{2}}\max\bigl\{ 1-\gamma,1-\gamma ^{-1}\bigr\} \bigl(\bigl\Vert \lambda^{k+1}- \lambda^{k}\bigr\Vert ^{2}-\bigl\Vert \lambda^{k}-\lambda^{k-1}\bigr\Vert ^{2}\bigr), \end{aligned}$$

where the inequality comes from $\alpha\in(0,1]$ and $\alpha\geq\frac {5-\varrho}{5}$. Based on (23), we can prove the worst-case $\mathcal {O}(1/t)$ convergence rate in an ergodic sense of the P-ADMM. □

### Theorem 3.1

*Suppose that Assumptions* 2.1-2.3 *and Assumption* 3.1 *hold*. *Let* $\{(x^{k},\lambda^{k})\}=\{(x_{1}^{k},x_{2}^{k},\lambda^{k})\}$ *be the sequence generated by the P*-*ADMM and let* $\bar{x}^{t}=\frac{1}{t}\sum_{k=1}^{t}x^{k+1}$, *where* *t* *is a positive integer*. *Then*,

24 $$ \textstyle\begin{cases} \vert \theta(\bar{x}^{t})-\theta(x^{*})\vert \leq\frac{D}{t},\\ \Vert A\bar{x}^{t}-b\Vert \leq\frac{D}{t\Vert \lambda^{*}\Vert }, \end{cases} $$

*where* $(x^{*},\lambda^{*})=(x_{1}^{*},x_{2}^{*},\lambda^{*})$ *with* $\lambda^{*}\neq0$ *is a point satisfying the KKT conditions in* (5), *and* *D* *is a constant defined by*

25 $$\begin{aligned} D={}& \frac{1}{2}\bigl\Vert x^{1}-x^{*}\bigr\Vert _{G_{1}}^{2}+\frac{1}{2} \bigl\Vert x_{2}^{1}-x_{2}^{*}\bigr\Vert _{N}^{2}+\frac{1}{2}\bigl\Vert x_{2}^{0}-x_{2}^{1}\bigr\Vert _{\alpha\bar {G}_{2}+\beta(1-\alpha)A_{2}^{\top}A_{2}}^{2} \\ &{} +\frac{1}{\beta\gamma}\bigl(\bigl\Vert \lambda^{1}\bigr\Vert ^{2}+\Vert \lambda \Vert ^{2}\bigr) +\frac{1}{2\beta\gamma^{2}} \max\bigl\{ 1-\gamma,1-\gamma^{-1}\bigr\} \bigl\Vert \lambda ^{1}-\lambda^{0}\bigr\Vert ^{2}. \end{aligned}$$

### Proof

Setting $x=x^{*}$ in the inequality (22) and summing it over $k= 1, 2,\ldots,t$, we obtain

$$\begin{aligned} &\sum_{k=1}^{t} \bigl[ \theta \bigl(x^{k+1} \bigr)-\theta \bigl(x^{*} \bigr)- \bigl\langle Ax^{k+1}-b,\lambda \bigr\rangle \ \bigr] \\ &\quad\leq\frac{1}{2} \bigl\Vert x^{1}-x^{*} \bigr\Vert _{G_{1}}^{2}+\frac{1}{2} \bigl\Vert x_{2}^{1}-x_{2}^{*} \bigr\Vert _{N}^{2}+\frac{1}{2} \bigl\Vert x_{2}^{0}-x_{2}^{1} \bigr\Vert _{\alpha\bar {G}_{2}+\beta(1-\alpha)A_{2}^{\top}A_{2}}^{2}\\ &\qquad{}+\frac{1}{2\beta\gamma} \bigl\Vert \lambda ^{1}-\lambda \bigr\Vert ^{2} \\ &\qquad{}+\frac{1}{2\beta\gamma^{2}}\max \bigl\{ 1-\gamma,1- \gamma^{-1} \bigr\} \bigl\Vert \lambda^{1}- \lambda^{0} \bigr\Vert ^{2} \\ &\quad\leq\frac{1}{2} \bigl\Vert x^{1}-x^{*} \bigr\Vert _{G_{1}}^{2}+\frac{1}{2} \bigl\Vert x_{2}^{1}-x_{2}^{*} \bigr\Vert _{N}^{2}+\frac{1}{2} \bigl\Vert x_{2}^{0}-x_{2}^{1} \bigr\Vert _{\alpha\bar {G}_{2}+\beta(1-\alpha)A_{2}^{\top}A_{2}}^{2}\\ &\qquad{}+\frac{1}{\beta\gamma} \bigl( \bigl\Vert \lambda ^{1} \bigr\Vert ^{2}+\Vert \lambda \Vert ^{2} \bigr) \\ &\qquad{}+\frac{1}{2\beta\gamma^{2}}\max \bigl\{ 1-\gamma,1- \gamma^{-1} \bigr\} \bigl\Vert \lambda^{1}- \lambda^{0} \bigr\Vert ^{2} , \end{aligned}$$

which together with the convexity of the function $\theta(\cdot)$ implies

$$\begin{aligned} &\theta \bigl(\bar{x}^{t} \bigr)-\theta \bigl(x^{*} \bigr)- \bigl\langle A \bar{x}^{t}-b,\lambda \bigr\rangle \\ &\quad\leq\frac{1}{2t} \bigl\Vert x^{1}-x^{*} \bigr\Vert _{G_{1}}^{2}+\frac{1}{2t} \bigl\Vert x_{2}^{1}-x_{2}^{*} \bigr\Vert _{N}^{2}+\frac{1}{2t} \bigl\Vert x_{2}^{0}-x_{2}^{1} \bigr\Vert _{\alpha\bar {G}_{2}+\beta(1-\alpha)A_{2}^{\top}A_{2}}^{2}\\ &\qquad{}+\frac{1}{\beta\gamma t} \bigl( \bigl\Vert \lambda ^{1} \bigr\Vert ^{2}+\Vert \lambda \Vert ^{2} \bigr) \\ &\qquad{}+\frac{1}{2\beta\gamma^{2} t}\max \bigl\{ 1-\gamma,1- \gamma^{-1} \bigr\} \bigl\Vert \lambda^{1}- \lambda^{0} \bigr\Vert ^{2} . \end{aligned}$$

Using the Lemmas 2.2 and 2.3 of [17] with $\rho=2\Vert \lambda^{*}\Vert $ (*ρ* is a parameter defined in Lemmas 2.2 and 2.3 of [17]), we can get (24). This completes the proof. □

### Remark 3.4

From (24) and (25), we can conclude that larger values of *γ* is more beneficial for accelerating the convergence of the P-ADMM, as the larger *γ*, the smaller *D*, which controls the upper bounds of $\vert \theta(\bar{x}^{t})-\theta(x^{*})\vert $ and $\Vert A\bar{x}^{t}-b\Vert $.

## Numerical experiments

In this section, we apply the P-ADMM to solve the compressive sensing, a concrete problem of the general model (1). The codes were written by Matlab R2010a and conducted on a ThinkPad notebook with Pentium(R) Dual-Core CPU T4400@2.2 GHz, 2 GB of RAM using Windows 7.

Let us briefly review the compressive sensing. Compressive sensing (CS) is to recover a sparse signal $\bar{x}\in\mathcal{R}^{n}$ from an undetermined linear system $y=A\bar{x}$, where $A\in\mathcal {R}^{m\times n}(m\ll n)$ is a linear mapping and $y\in\mathcal{R}^{m}$ is an observation. An important decoding model of CS is

26 $$ \min_{x\in\mathcal{R}^{n}}\frac{1}{2}\Vert Ax-y\Vert ^{2}+\nu \Vert x\Vert _{1}, $$

where the parameter $\nu>0$ is used to trade off both terms for minimization. This is a special case of the general two-block separable convex minimization model (1). In fact, setting $x_{1}=x, x_{2}=x$, (26) can be recast as

$$\begin{aligned} &\min\frac{1}{2}\Vert Ax_{1}-y\Vert ^{2}+\nu \Vert x_{2}\Vert _{1} \\ &\mathrm{s.t.}\ x_{1}-x_{2}=0, \\ &\quad x_{1}\in\mathcal{R}^{n}, x_{2}\in \mathcal{R}^{n}, \end{aligned}$$

which is a special case of (1) with

$$\begin{aligned} &\theta_{1}(x_{1})=\frac{1}{2}\Vert Ax_{1}-y\Vert ^{2},\qquad \theta_{2}(x_{2})= \nu \Vert x_{2}\Vert _{1},\\ & A_{1}=I_{n},\qquad A_{2}=-I_{n},\qquad b=0,\qquad\mathcal{X}_{1}= \mathcal{X}_{2}=\mathcal{R}^{n}, \end{aligned}$$

and thus, the P-ADMM can be used to solve CS.

In our experiment, the stopping criterion of the P-ADMM is set as

$$\frac{\Vert f_{k}-f_{k-1}\Vert }{\Vert f_{k-1}\Vert }< 10^{-5}, $$

where $f_{k}$ denotes the function value of (26) at the iterate $x_{1}^{k}$. The initial points of $x_{1},x_{2},\lambda$ are all set as $A^{\top}y$, and due to the limit of EMS memory of our computer, we only test a medium scale of (26) with $n=1{,}000, m=300$, $k=60$, where *k* is the number of random nonzero elements contained in the original signal. In addition, we set

$$\bar{A}=\operatorname{randn}(m,n), \qquad[Q, R]=\operatorname{qr} \bigl( \bar{A}',0 \bigr), \qquad A = Q', $$

and $\nu=0.01$, $G_{1}=\tau I_{n}-\beta A^{\top}A$ with $\tau=2, \beta =\operatorname{mean}(\operatorname{abs}(b))$. In the literature, the relative error (RelErr) is usually used to measure the quality of recovered signal and is defined by

$$\operatorname{RelErr}=\frac{\Vert \tilde{x}-\bar{x}\Vert }{\Vert \bar{x}\Vert }, $$

where *x̃* and *x̄* denote the recovered signal and the original signal, respectively.

**Figure 1:**
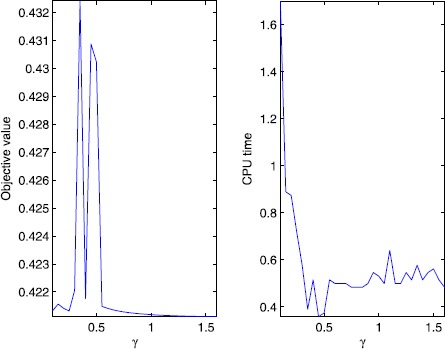
**Objective value and CPU time with different** ***γ*** **.**

**Figure 2:**
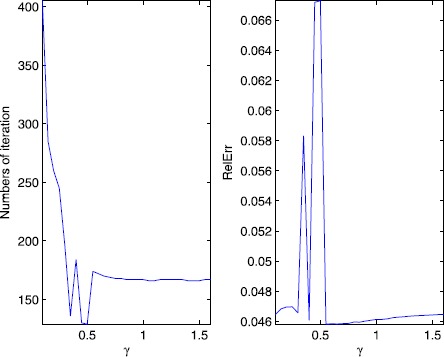
**Numbers of iteration and relative error with different** ***γ*** **.**

First, let us illustrate the sensitivity of *γ* for the P-ADMM. We choose different values of *γ* in the interval [0.1, 1.6] (More specifically, we take $\gamma =0.10,0.15,\ldots,1.60$). The numerical results of the objective value of (26) and the CPU time in seconds requited by the P-ADMM are depicted in Figure 1, and the numerical results of the numbers of iteration and the RelErr required by P-ADMM are depicted in Figure 2.

According to the curves in Figures 1-2, we can see that the relaxation factor *γ* works well for a wide range of values and, based on this experiment, the values greater than 0.5 are more preferred.

**Figure 3:**
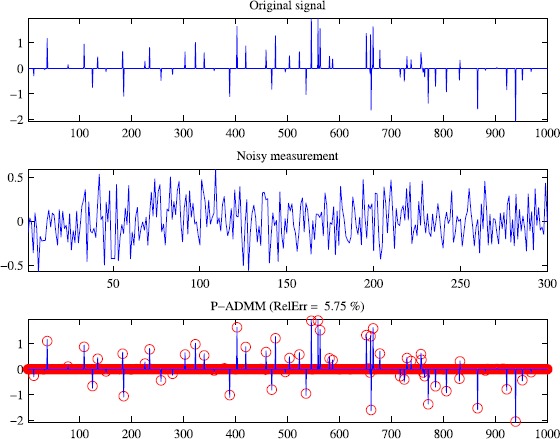
**The original signal, noisy measurement and recovered results.**

Now let us test the effectiveness of the P-ADMM with the indefinite proximal matrix $G_{2}=(\alpha\tau-\beta)I_{n}$. Here we set $\alpha=0.8, \tau=1.1$, $\beta=1$, and $\gamma=1$. The numerical results of one experiment are as follows: the objective value is 0.4291; the CPU time is 1.0920; the numbers of iteration is 378 and the RelErr is 5.75%. The original signal, the measurement and the signal recovered by the P-ADMM for this test scenario are given in Figure 3. The recovered results are marked by a red circle in the third subplot of Figure 3, which shows clearly that almost the original signal is recovered with high precision. This indicates that the P-ADMM is effective though the proximal matrix $G_{2}$ is indefinite.
